# Application of spectrophotometry in novel simultaneous dissolution profiling of a single pill triple therapy of amlodipine, perindopril and indapamide; whiteness evaluation

**DOI:** 10.1186/s13065-025-01396-3

**Published:** 2025-02-07

**Authors:** Aya T. Soudi

**Affiliations:** Analytical Chemistry Department, Faculty of Pharmacy, Cairo University, Kasr El-Aini Street, Cairo, 11562 Egypt

**Keywords:** Amlodipine besylate, Perindopril arginine and indapamide, Derivative spectrophotometry, Ratio difference technique, Dual wavelength method, Dissolution testing, Whiteness evaluation

## Abstract

Simple, diverse univariate spectrophotometric methods were developed and validated for the determination of amlodipine besylate (AM), perindopril arginine (PE), and indapamide (ID). The first method involved direct measurement of AM absorbance at 365 nm within a concentration range of 2.00–40.00 µg/mL, where PE and ID exhibited no spectral interference. To eliminate the contribution of AM from the ternary mixture, its spectrum was divided by a reference spectrum of AM (12 µg/mL), followed by mathematical subtraction of the resulting constant. The spectrum was then multiplied by the AM divisor to yield a corrected spectrum of the PE and ID binary mixture, allowing their quantification. Various approaches were used to quantify both drugs, including measurement of their second (2DD) and first derivative (1DD) spectra at 231.30 nm and 251.00 nm, respectively. Additionally, the ratio difference (RD) technique and dual wavelength (DW) method were employed. The concentration ranges for PE and ID were 5.00–100.00 µg/mL and 1.00–20.00 µg/mL, respectively. Among these methods, the DW technique was the simplest, so it was chosen for dissolution monitoring of PE and ID. These methods were successfully applied to determine AM, PE, and ID in bulk powder, as well as in Triplixam^®^ tablets, without interference from excipients and in different used dissolution media. The whiteness of the method was evaluated, demonstrating its excellent environmental, analytical and practical efficiency.

**Supplementary Information:**

The online version contains supplementary material available at 10.1186/s13065-025-01396-3.

## Introduction

**Fig. 1:**
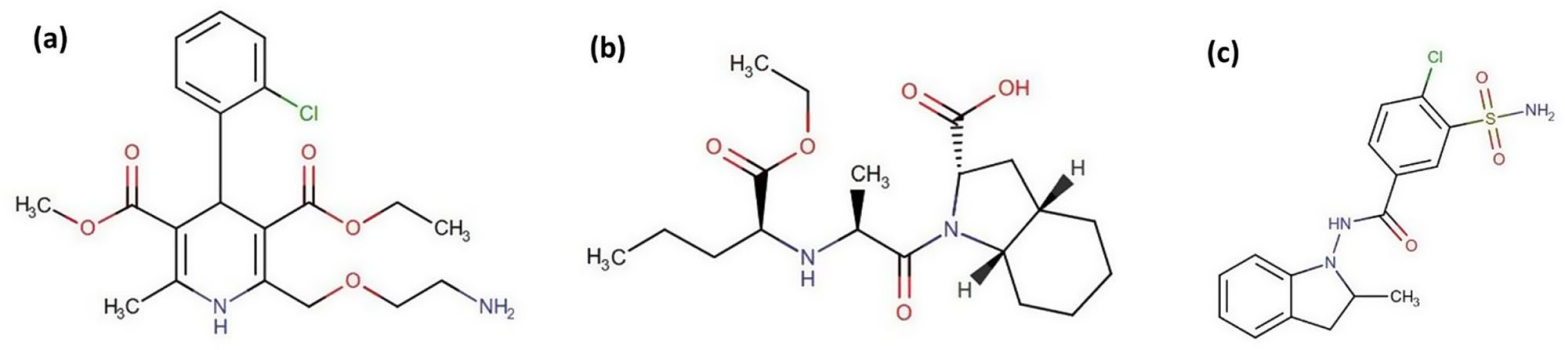
Chemical structure of (**a**) AM, (**b**) PE, and (**c**) ID

Oral dosage forms continue to be one of the most complying formulations available to patients. Their effectiveness depends upon the drug ability to dissolve in the gastrointestinal tract fluids before their absorption into the circulation. Therefore, the rate of drug dissolution from tablets is pivotal. Dissolution testing is very important in adding in-vivo relevance to an in-vitro data which provide a realistic correlation between in-vitro/in-vivo results. In quality control laboratories, dissolution studies are performed to assess the quality of the dosage form and to evaluate the newly developed drug products [1]. FDA describes a dissolution method for tablets containing Amlodipine besylate (AM), Perindopril arginine (PE) and Indapamide (ID) separately, and for tablets containing a mixture of AM and PE [2]. In the present research, we are dealing with Triplixam^®^ tablets, which are a combination of three active ingredients: AM, PE and ID. Each active ingredient helps lower blood pressure, and together they effectively manage hypertension. Chemically; AM is 3-Ethyl 5-methyl (4*RS*)-2-[(2-aminoethoxy)methyl]-4-(2-chlorophenyl)-6-methyl-1,4- dihydropyridine-3,5-dicarboxylate benzenesulfonate [3] (Fig. 1a). AM is a widely prescribed medication that falls under the category of calcium channel blockers. It is commonly utilized to manage hypertension, angina, and myocardial ischemia [4]. PE is 2-Methylpropan-2-amine (2*S*,3a*S*,7a*S*)-1-[(2*S*)-2-[[(1*S*)-1-(ethoxycarbonyl)butyl]amino] propanoyl]octahydro-1*H*-indole-2-carboxylate [3] (Fig. 1b). As an angiotensin-converting enzyme (ACE) inhibitor, PE blocks the conversion of angiotensin I to angiotensin II, a crucial component of the renin-angiotensin-aldosterone system. It is prescribed for managing mild to moderate essential hypertension and congestive heart failure [4]. ID is 4-Chloro-*N*-[(2*RS*)-2-methyl-2,3-dihydro-1*H*-indol-1-yl]-3-sulfamoylbenzamide [3] (Fig. 1c). ID is a diuretic used to treat hypertension, either alone or in combination with other antihypertensive medications. It is also employed to manage salt and fluid retention related to congestive heart failure [5].

Searching the literature, we found one Thin layer chromatographic method (TLC) [6], one LC-MS/MS method [7], three HPLC methods [8–10] for simultaneous determination of the ternary mixture. In addition to one ultraviolet absorbance correction method [11], however it shows no real application on dosage forms or in any dissolution mediums. Spectrophotometric techniques are proved to be direct, quick, and less expensive than any other methods for the determination of drugs in mixture form in different pharmaceutical formulations. So, the target of this work was to establish, and validate different simple univariate spectrophotometric methods for determination of the three drugs in a single pill dosage form. Furthermore, dissolution testing of the three drugs was done simultaneously which open a new era for spectrophotometric methods to be a real-world application extending their use beyond just drug determination or dosage form quantification.

In recent years, Green Analytical Chemistry (GAC) has become a global trend among analysts aiming to develop environmentally sustainable analytical methods. The focus is on minimizing the use of harmful solvents and reducing waste while ensuring safer and less toxic procedures [12, 13]. However, improving a method’s greenness should not come at the cost of its performance. To address this, White Analytical Chemistry (WAC) was introduced as an extension of GAC. WAC ensures a balanced approach, incorporating environmental, analytical, and practical considerations to ensure both functionality and sustainability. The “whiteness” of a method is measured by evaluating it against the 12 principles of WAC, making it a useful tool for selecting and comparing the best analytical methods [14]. By integrating the principles of WAC with our spectrophotometric approach, we provide a robust, eco-friendly solution for analyzing AM, PE, and ID in both their combined pharmaceutical dosage form and their dissolution medium.

## Experimental

### Instrument and software

The analysis was conducted using a SHIMADZU UV-1650 double-beam UV-visible spectrophotometer (Kyoto, Japan), equipped with UVProbe software version 2.21 and connected to an IBM-compatible PC and an HP 1020 LaserJet printer. Measurements were performed with two matched 10-mm quartz cells. The instrument was set with a spectral bandwidth of 2 nm, a scanning speed of 2800 nm/min, and an interval of 0.1 nm.

### Materials and reagents

#### Pure standards and pharmaceutical formulation

Pfizer, Cairo, Egypt, kindly provided the AM Standard certified to contain 99.8%. Laboratoires Servier, Cairo, Egypt, supplied the PE and ID standards certified to contain 99.7% and 99.5%, respectively.

Triplixam^®^ tablets - produced by Laboratoires Servier – are labeled to contain: 13.87 mg of AM, 10 mg of PE, and 2.5 mg of ID were procured from the gulf region.

#### Reagents and chemicals

Analytical grade hydrochloric acid was sourced from El-Nasr Pharmaceutical Chemicals Company in Cairo, Egypt. Potassium dihydrogen phosphate and disodium hydrogen phosphate were obtained from Adwic, also located in Cairo, Egypt. Ultra-pure water (18 Mohm·cm) was generated using an Elga Ultrapure Q system. Spectroscopic grade methanol was acquired from Sigma Aldrich in Cairo, Egypt.

Phosphate buffer solution at pH 6.8 and 0.01 M HCl were prepared using ultra- pure water.

The reagents used in the reference HPLC method are as follows: Phosphate buffer with pH 2, containing decane sulphonate as an ion-pairing agent (Reagent A), and acetonitrile (Reagent B).

### Standard solutions

Stock standard solutions of 1 mg/mL for AM, PE, and ID were prepared by accurately weighing 50.0 mg of each compound and dissolving them in separate 50-mL volumetric flasks with methanol to achieve the desired volume.

Working standard solutions were then prepared as follows: 200 µg/mL AM, 200 µg/mL PE, and 100 µg/mL ID. These solutions were made in both 0.01 M HCl and phosphate buffer at pH 6.8.

### Procedures

#### Spectral characteristics

The zero-order (0D) absorption spectra of AM, PE, and ID (at concentrations of 15, 20, and 10 µg/mL, respectively) were recorded using 0.01 M HCl as the blank.

#### Calibration curves development

##### Direct method (_0_D) for AM determination

Accurate aliquots of the AM working standard solution were transferred into a series of 10-mL volumetric vials and diluted with 0.01 M HCl to achieve concentrations ranging from 2.00 to 40.00 µg/mL. The zero-order absorption spectrum of each solution was then recorded using 0.01 M HCl as the blank.

The recorded absorbance values at 365 nm - where there is no contribution from PE and ID - were plotted against AM concentration in µg/mL and the regression equation of the calibration curve was computed.

##### Removing AM contribution

AM contribution in the ternary mixture spectrum can be omitted by dividing the mixture spectrum with the spectrum of AM standard (12.00 µg/mL). Posteriorly, the resulting constant is then subtracted, and the spectrum is subsequently multiplied by the AM standard spectrum. This leads to a recovered spectrum representing a binary mixture of PE and ID. The concentrations of both components can be determined by derivative spectrophotometry, ratio difference technique and dual wavelength method.

##### Derivative spectrophotometry (DD)

For PE determination, the peak amplitude of the second derivative (^2^DD) of the recovered binary mixture spectrum was measured at 231.3 nm using Δ λ = 4 and a scaling factor = 1000.

For ID determination, the peak amplitude of the first derivative (^1^DD) of the recovered binary mixture spectrum was measured at 251 nm using Δ λ = 2 and a scaling factor = 10.

A linear correlation was established between the peak amplitudes of the second derivative for PE and the first derivative for ID against their respective concentrations. The regression equations for these relationships were then calculated.

##### Ratio difference technique (RD)

For the determination of PE, the difference in peak amplitudes (ΔP) of the ratio spectra (PE/ID 5 µg/mL) at 219 and 267 nm (ΔP219–267) was plotted against the corresponding PE concentrations in µg/mL, and the regression equation was derived. Similarly, to determine ID, the difference in peak amplitudes of the ratio spectra (ID/PE 70 µg/mL) at 219 and 248 nm (ΔP248–219) was plotted against the corresponding ID concentrations in µg/mL, followed by the calculation of the regression equation.

##### Dual wavelength method (DW)

For PE determination, the absorbance difference (ΔA) of the recovered binary mixture spectra at 218 and 244 nm (ΔA 218–244) was calculated and plotted versus PE corresponding concentrations where the difference in ID spectrum is zero.

For ID determination, the absorbance difference (ΔA 254–350) from the recovered binary mixture spectra was measured and plotted against the corresponding concentrations of ID where the difference in PE spectrum is zero.

From the constructed calibration curves, regression equations were computed for PE and ID.

#### Laboratory made-up mixtures analysis

Various laboratory-prepared mixtures with different ratios of AM, PE, and ID were created, scanned, and saved. The procedures outlined under " calibration curves development” were followed to accurately determine the concentrations of each of the three drugs.

#### Determination of AM, PE and ID in Triplixam® tablets using the proposed methods

Ten Triplixam^®^ tablets were accurately weighed and crushed into a fine powder. A portion of the powder, equivalent to the content of one tablet, was transferred to a 100-mL volumetric flask. To dissolve the sample, 50 mL of methanol was added, and the mixture was sonicated for 20 min. The volume was then brought to the 100-mL mark with methanol, followed by filtration. A 1 mL aliquot of the filtrate was transferred into a 10-mL volumetric flask and diluted with 0.01 M HCl to obtain final concentrations of 13.87 µg/mL for AM, 10 µg/mL for PE, and 2.5 µg/mL for ID. The concentrations of AM, PE, and ID were determined using the methods described in the “calibration curves development” section.

#### Dissolution monitoring of AM, PE and ID in Triplixam® tablets

VanKel VK 7000 USP II apparatus was used in dissolution monitoring of the pills. The apparatus consists of six vessels each with 500 mL of the dissolution medium thermostatically set at 37 ± 0.5 °C. The medium was whiskered using a Teflon coated paddle at 75 rpm rotation rate. Two different dissolution mediums were separately used: 0.01 M HCl and phosphate buffer solution at pH 6.8. Samples were taken from each dissolution medium at 5, 10, 15, 20, 30, 45, and 60-minute intervals, filtered, and scanned. The concentration of AM was determined directly by measuring its zero-order absorbance at 365 nm. Then, AM contribution was removed as mentioned before under “calibration curves development”. On the recovered binary mixture spectrum, DW method was applied to get PE and ID concentrations. Percentage dissolution was then calculated for the three drugs either in 0.01 M HCl or phosphate buffer pH 6.8 mediums. Two dissolution curves for Triplixam^®^ were plotted.

## Results and discussion

Univariate spectrophotometric resolution techniques are one of the simplest analytical methods to develop and validate. These techniques are known for their high sensitivity, precision and versatility where, they can be used in analysis of different multi-components formulations [15–20]. Spectrophotometric methods and High-performance liquid chromatographic methods (HPLC) are the official methods used for dissolution testing of different dosage forms [21]. For all solid oral dosage forms, dissolution testing is essential and is applied during the manufacturing of drug products and also during stability testing to assess the quality of the manufactured drug products [1, 22]. A critical challenge in pharmaceutical formulation development is ensuring that drug levels in the body are properly adjusted to achieve the desired therapeutic effect. Failure to maintain the drug concentration within the therapeutic window can result in insufficient bioavailability, leading to reduced efficacy, or excessive bioavailability, which may cause harmful toxic effects. The dissolution test helps to know the rate of the release of the active pharmaceutical ingredient from the dosage form [22]. Here, we addressed Triplixam^®^ tablets as a multi-component formulation of AM, PE and ID. There is no previous official nor reported methods were found for simultaneous dissolution monitoring of this combination. Various simple univariate methods are employed to analyze the three drugs in both their powdered form and in pharmaceutical dosage forms. Additionally, dissolution monitoring of these drugs is simultaneously conducted using two different dissolution media. The FDA outlined specific dissolution testing protocols for tablets containing AM alone, AM with PE, and ID alone [2]. Tablets containing AM alone and AM with PE were tested in 0.01 M HCl, while those containing ID utilized phosphate buffer pH 6.8. Therefore, we used these two media to observe the dissolution behavior of Triplixam^®^ tablets.

**Fig. 2:**
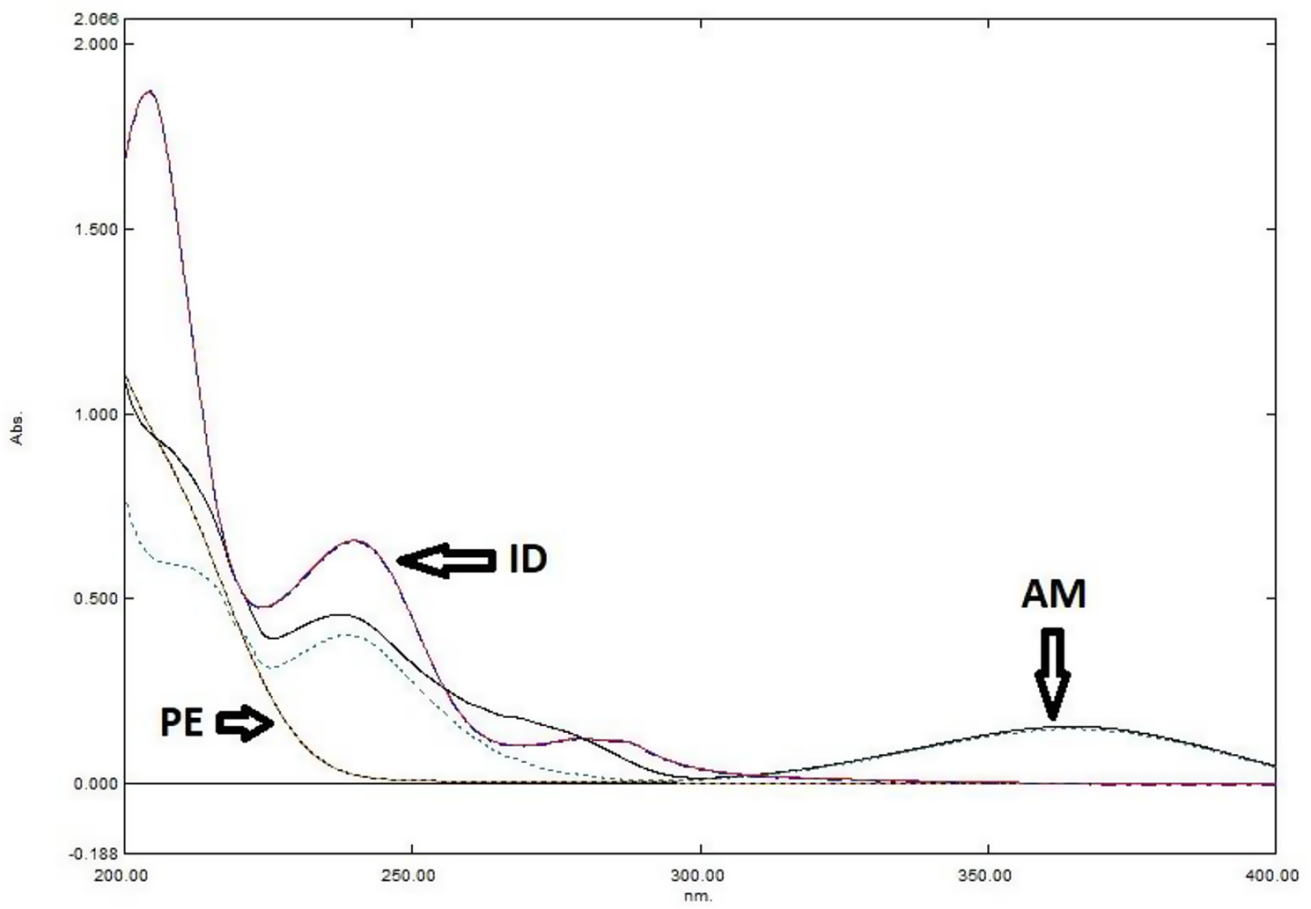
Zero order shows that PE (50 µg/mL) and ID (10 µg/mL) are superimposed in 0.01 M HCl and phosphate buffer pH 6.8 but AM (12 µg/mL) is not

**Fig. 3:**
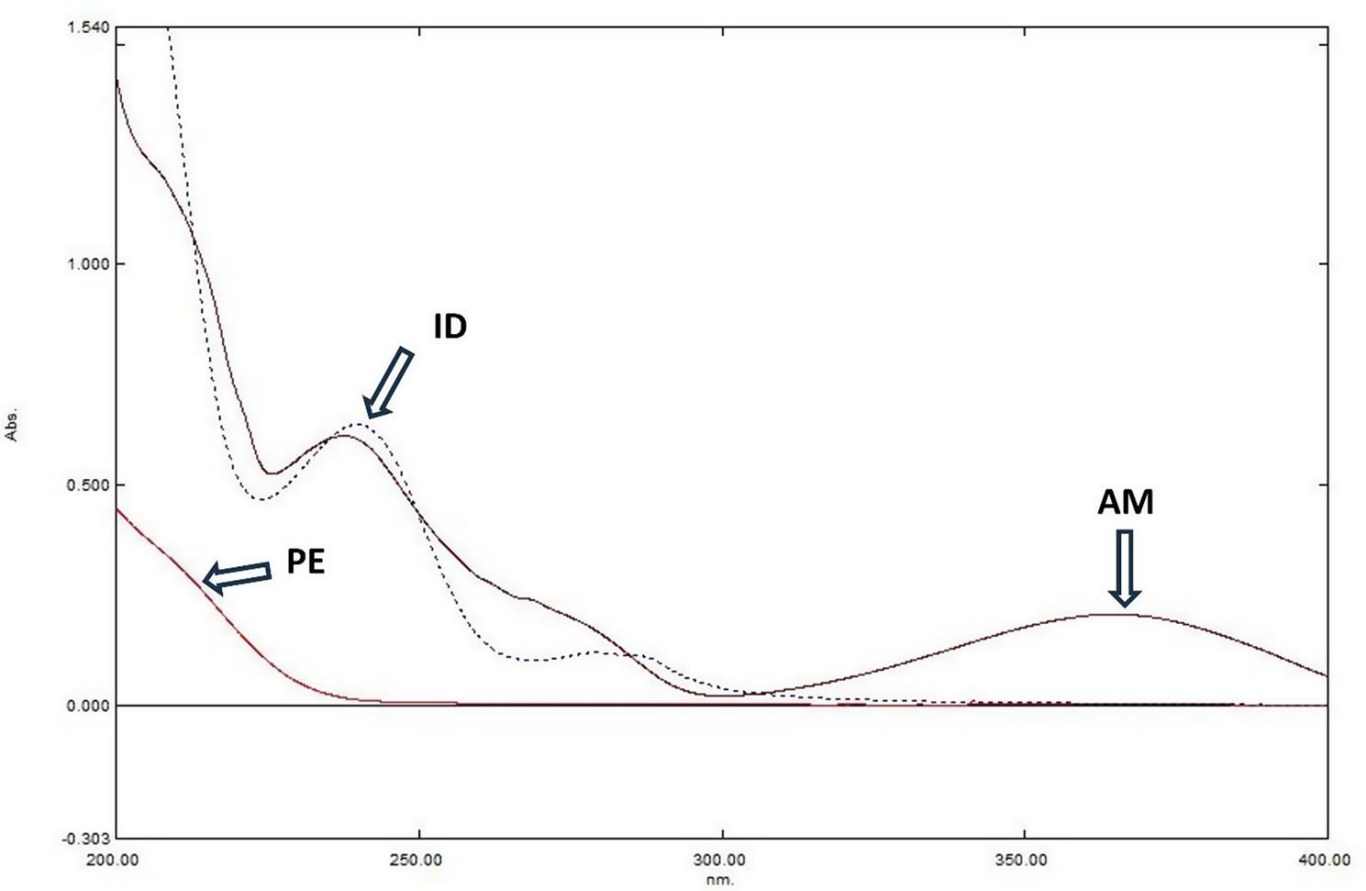
Zero order (^0^D) absorption spectra of AM (15 µg/mL), PE (20 µg/mL) and ID (10 µg/mL)

AM had different spectra in phosphate buffer (pH 6.8) compared to 0.01 M HCl, but PE and ID have a superimposed spectra in both solvents (Fig. 2). The zero order (^0^D) spectra of AM, PE and ID are presented in Fig. 3. The overlap in the absorption spectra hinders the direct resolution of the ternary mixture, except for AM, which has a sufficiently extended spectrum to allow its direct determination in the presence of PE and ID.

### Direct method (^0^D) for AM determination

AM had different spectra in 0.01 M HCl and in phosphate buffer pH 6.8, but in both situations, it is extended over PE and ID. SO, it can be directly determined at 365 nm without any contribution from PE and ID.

Various laboratory-prepared mixtures were analyzed, and the concentration of AM was determined by measuring its absorbance at 365 nm. The corresponding concentration was then calculated using the regression equation (Figure S1).

The contribution of AM was eliminated by dividing the mixture’s spectrum by the divisor spectrum of AM (12 µg/mL) prepared in the same solvent. The resulting constant was then subtracted mathematically, and the spectrum was multiplied by the divisor again. This process effectively removed AM’s spectrum, leaving behind the spectrum of the binary mixture of PE and ID, allowing for the application of various methods to quantify both components.

After AM removal and owing to that PE and ID spectra are superimposed in both phosphate buffer pH 6.8 and 0.01 M HCl, all the next procedures will be done in 0.01 M HCl only.

### Derivative spectrophotometry (DD)

**Fig. 4:**
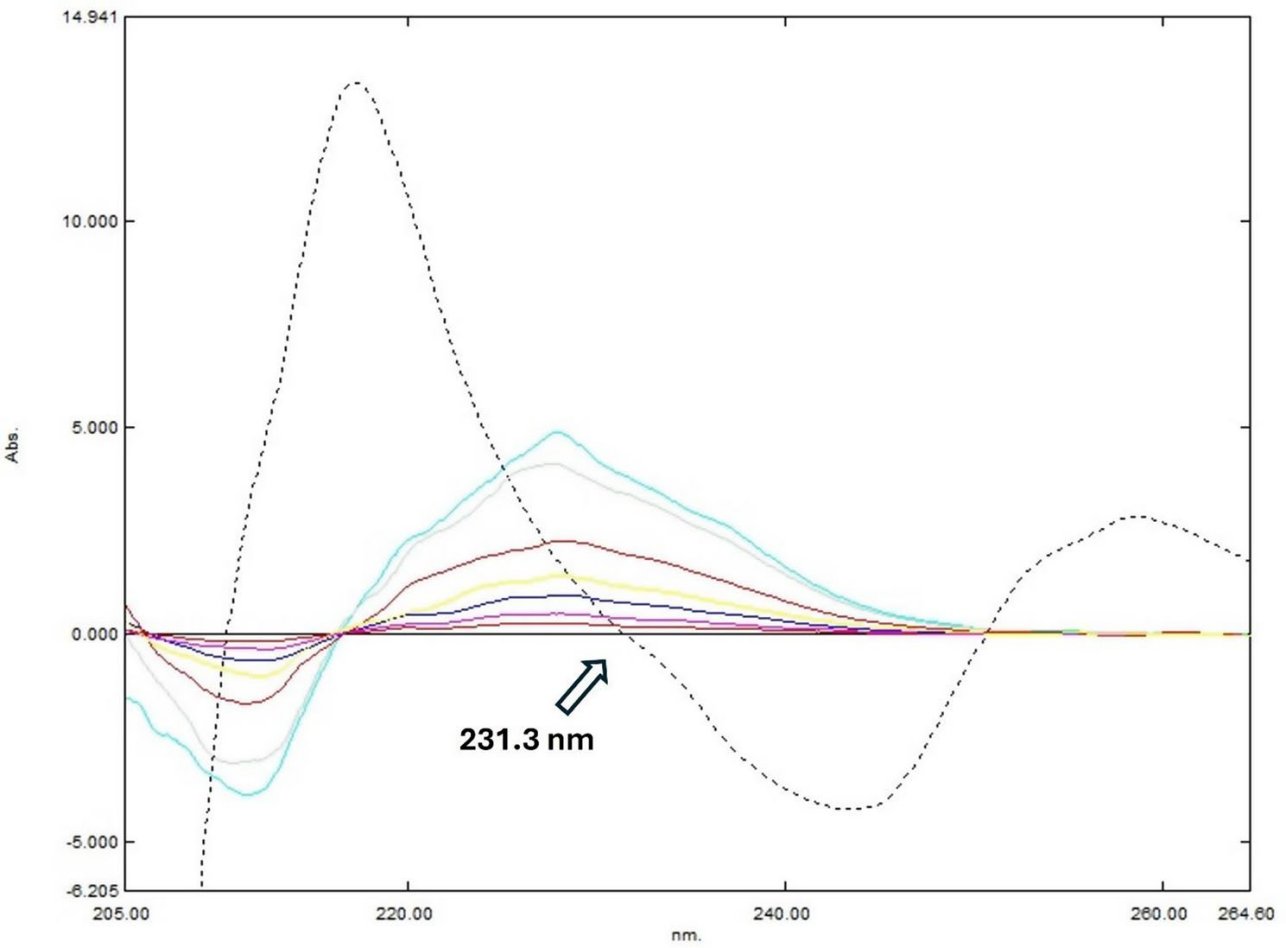
Second derivative calibration of PE at 231.3 nm at the zero crossing of ID, showing spectra for individual concentrations of 5, 10, 20, 30, 50, 90, and 100 µg/mL

**Fig. 5:**
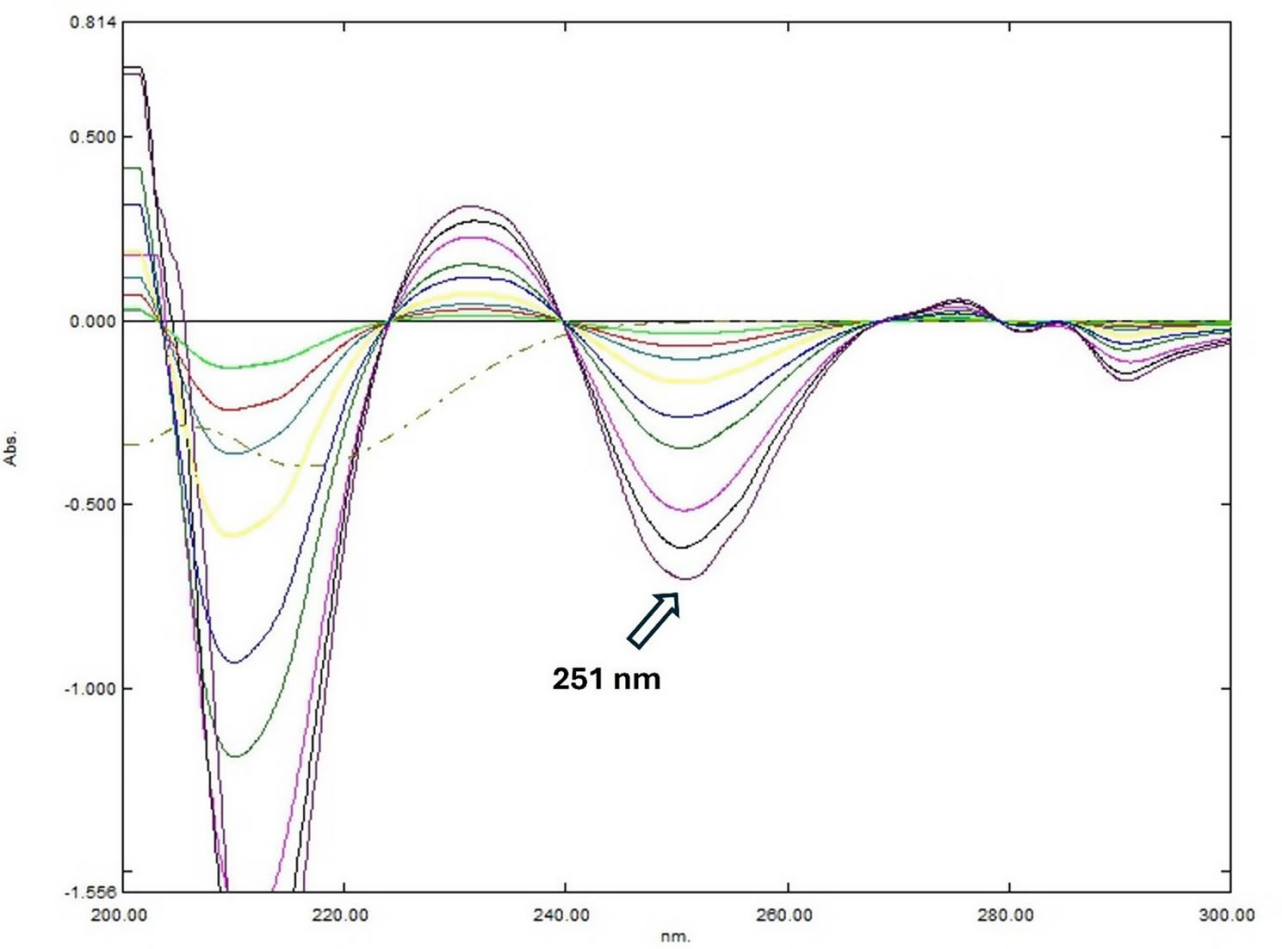
First derivative calibration of ID (1–20 µg/mL) at 251 nm at zero contribution of PE

Derivative resolution technique uses simple spectra manipulation to determine certain drug concentration without any interference from other components in the same mixture. The concentration of PE can be determined by applying the second derivative (^2^DD) to the recovered binary mixture spectrum, using Δλ of 4 and a scaling factor of 1000 at 231.3 nm, where ID shows zero interference (Fig. 4). Similarly, the concentration of ID can be measured by applying the first derivative (^1^DD) to the same spectrum with a Δλ of 2 and a scaling factor of 10 at 251 nm, where PE exhibits no contribution (Fig. 5).

Laboratory made-up mixtures were analyzed for PE and ID concentrations, where both drugs can be accurately obtained from their corresponding regression equations.

### Ratio difference technique (RD)

Both PE and ID were quantified using the two-step ratio difference method. Different PE and ID divisor concentrations spectra were tried to determine ID and PE, respectively. The chosen divisors should be selected to give the maximum sensitivity and minimal noise. Optimal results were obtained using divisor concentrations of 70 µg/mL for PE and 5 µg/mL for ID.

**Fig. 6:**
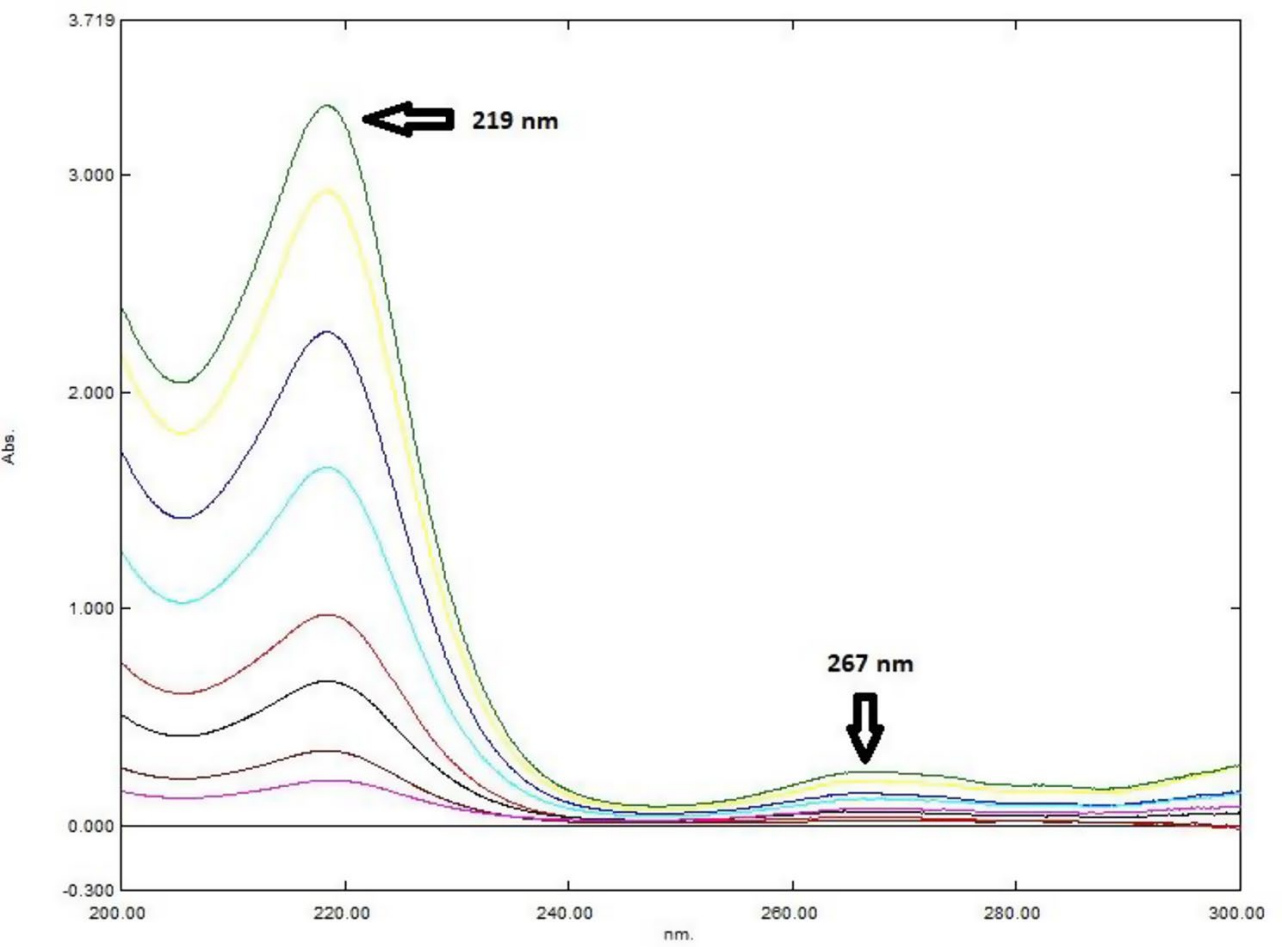
Ratio spectra of (5–100 µg/mL) PE using the spectrum of 5 µg/mL ID as divisor

**Fig. 7:**
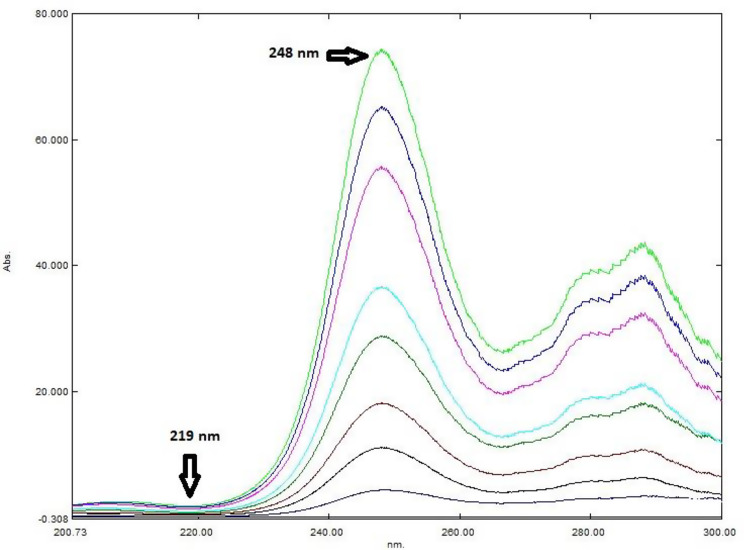
Ratio spectra of (1–20 µg/mL) ID using the spectrum of 70 µg/mL PE as divisor

The recovered binary mixture spectrum was first divided by the spectrum of 5 µg/mL ID, and the difference in peak amplitudes (ΔP) at 219 and 267 nm (ΔP219–267) was calculated to determine the concentration of PE using its corresponding regression equation (Fig. 6). Similarly, the recovered binary mixture spectrum was divided by the spectrum of 70 µg/mL PE, and the difference in peak amplitudes (ΔP) at 219 and 248 nm (ΔP248–219) was calculated to determine the concentration of ID based on its respective regression equation (Fig. 7).

Laboratory made-up mixtures were analyzed for PE and ID concentrations, where both drugs can be accurately obtained from their corresponding regression equations.

### Dual wavelength method (DW)

The DW method calculates concentrations of both PE and ID by applying a single mathematical operation on the recovered binary mixture spectrum, without requiring a divisor like the RD method or any additional manipulation steps as in derivative spectrometry.

**Fig. 8:**
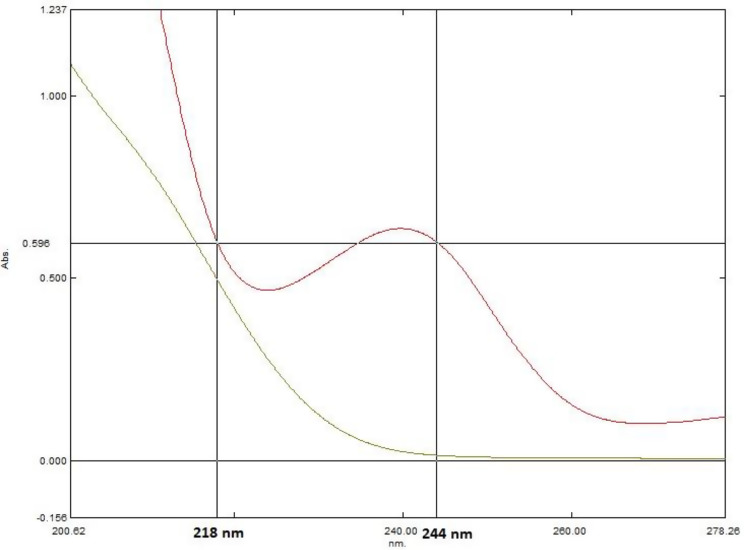
The zero-order absorption spectra of PE and ID indicate equal absorbance for ID and differing absorbance for PE at 218 and 244 nm for PE determination using the dual wavelength method

To determine PE, two wavelengths (218 and 244 nm) were chosen where ID absorbs equally, but PE shows a difference in absorbance (Fig. 8). Therefore, the absorbance difference (ΔA = 218–244) in the recovered binary mixture spectrum correlates solely with PE concentration.

**Fig. 9:**
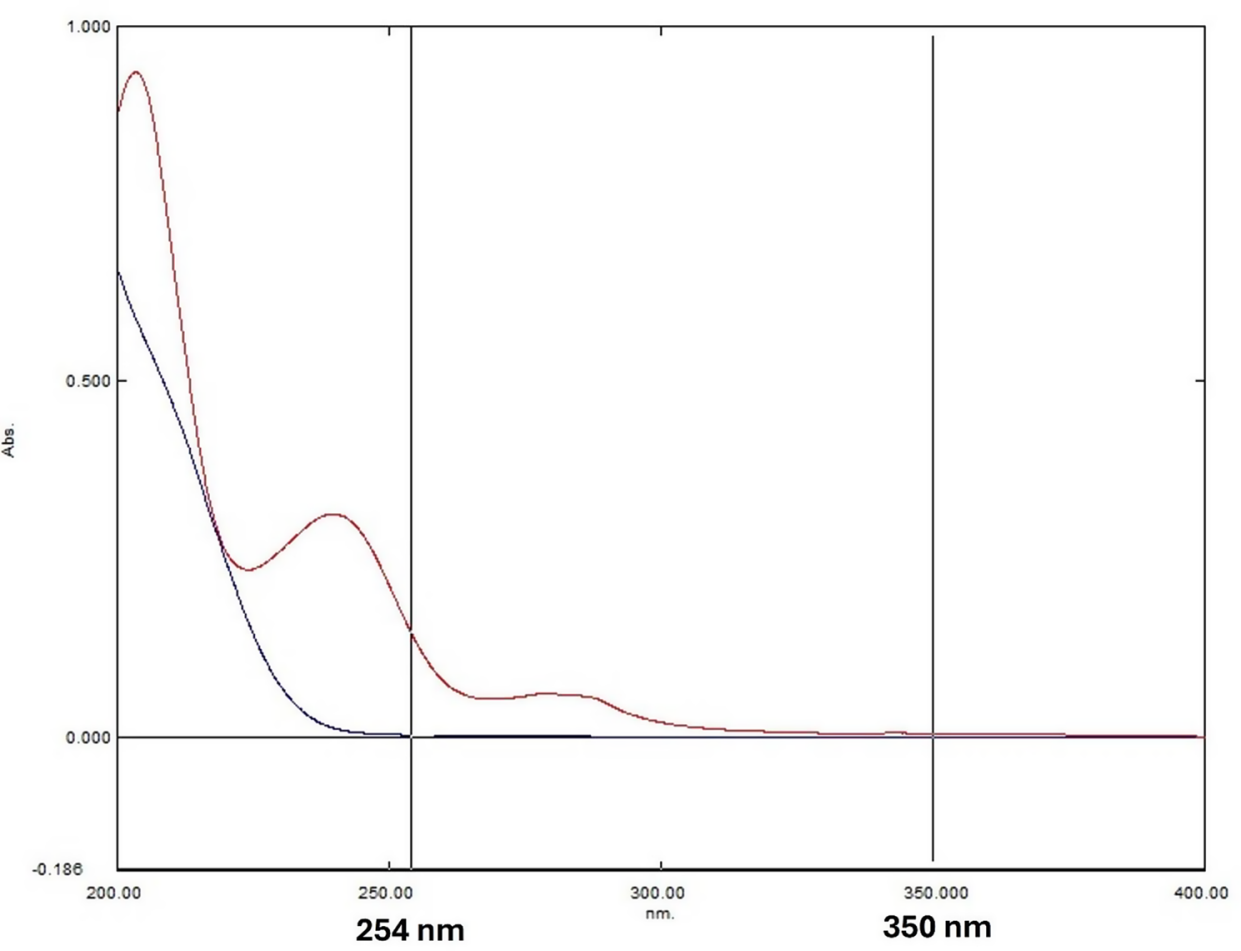
The zero-order absorption spectra of PE and ID indicate equal absorbance for PE and differing absorbance for ID at 254 and 350 nm for ID determination using the dual wavelength method

To determine ID, wavelengths (254 nm and 350 nm) were chosen where PE absorbs equally, but ID exhibits a difference in absorbance (Fig. 9). Thus, the absorbance difference (ΔA 254–350) in the recovered binary mixture spectrum is solely indicative of ID concentration.

The absorbance difference Δ*A* values obtained from Laboratory-prepared mixtures are used to substitute in PE and ID obtained regression equations to determine their concentrations.

### Method validation

The proposed methods were validated following ICH guidelines [23]. Key parameters such as linearity, selectivity, precision, and accuracy were assessed, yielding satisfactory results as summarized in Table 1.

**Table 1:** Analytical parameters and Validation results for the determination of AM, PE and ID by the proposed spectrophotometric methods

Parameter	AM	PE			ID		
	Direct method	2nd derivative	RD	DW	1st derivative	RD	DW
Wavelength (nm)	365	231.3	219–267	218–244	251	248 − 219	254–350
**Regression parameters**							
Linearity range (µg/mL)	2–40	5–100	5–100	5–100	1–20	1–20	1–20
Intercept	0.0007	− 0.0231	0.0172	0.0032	− 0.0024	0.1242	− 0.0005
Slope	0.0128	0.0394	0.0302	0.0096	0.035	3.5967	0.0295
Correlation Coefficient	0.9998	0.9998	0.9998	0.9997	0.9998	0.9999	0.9998
**Accuracy (Mean ± RSD)** ^h^							
Low concentration^a^	98.89 ± 1.14	102.30 ± 0.25	100.82 ± 0.19	101.53 ± 0.59	101.52 ± 0.81	100.45 ± 0.01	101.41 ± 0.98
Medium concentration^b^	100.38 ± 0.22	100.51 ± 0.05	99.90 ± 0.04	100.10 ± 0.12	100.47 ± 0.22	101.63 ± 0.01	101.92 ± 0.45
High concentration^c^	101.11 ± 0.13	99.03 ± 0.016	99.12 ± 0.02	98.74 ± 0.07	100.69 ± 0.09	100.36 ± 0.01	99.85 ± 0.19
**Precision (±%RSD)** ^h^							
Repeatability^d^	± 0.35	± 0.08	± 0.35	± 0.22	± 0.26	± 0.02	± 0.73
Intermediate precision^e^	± 0.58	± 0.15	± 0.58	± 0.49	± 0.51	± 0.05	± 0.9
Selectivity^f, h^	100.90 ± 1.84	99.85 ± 1.87	101.90 ± 0.42	100.73 ± 1.30	99.34 ± 1.09	98.13 ± 0.95	99.39 ± 0.94
LOD^g^	0.62	1.55	1.63	1.39	0.33	0.25	0.30
LOQ^g^	1.89	4.72	4.94	4.20	1.00	0.76	0.90

a Accuracy low concentration (4, 10, and 2 µg/ mL) for AM, PE, and ID, respectively

b Accuracy medium concentration (20, 50, and 7.5 µg/ mL) for AM, PE, and ID, respectively

c Accuracy high concentration (35, 90, and 17.5 µg/ mL) for AM, PE, and ID, respectively

d Intraday precision (the %RSD of 3 different concentrations (8, 16, 40 µg/ mL for AM, 20, 70, 100 µg/ mL PE and 3, 10, 20 µg/ mL for ID)/3 replicates each, within the same day)

e Interday precision (the %RSD of 3 different concentrations (8, 16, 40 µg/ mL for AM, 20, 70, 100 µg/ mL PE and 3, 10, 20 µg/ mL for ID)/3 replicates each, repeated on 3 successive days)

f Mixture concentrations used for the selectivity study were 27.5/20/5, 12/40/10, 30/10/5, and 20/7/20 µg/mL for Amlodipine, Perindopril, and Indapamide, respectively

g Calculated from equation [LOD = 3.3 (S.D/S), LOQ = 10 (S. D/S); where S.D is the residual standard deviation of the slope and S is the slope for the proposed methods

h Acceptability criteria: Accuracy was deemed acceptable within ± 2%, precision with an RSD ≤ 2%, and selectivity confirmed by the absence of significant interference which proved by an RSD ≤ 2% for all tested mixtures

### Assay of AM, PE and ID in triplixam ® tablets

Using the suggested techniques, we determined the concentrations of AM, PE, and ID in Triplixam^®^ tablets, a multi-component formulation, achieving results that closely match the labeled values. The standard addition technique verified that there was no interference from the tablet’s inactive ingredients (Table 2). Moreover, statistical analysis comparing our methods with the reported HPLC method [8] revealed no significant differences, demonstrating the practical usefulness of our new methods for analyzing this pharmaceutical formulation.

**Table 2:** Determination of AM, PE and ID in Triplixam^®^ Tablets* by the proposed spectrophotometric methods and application of standard addition technique

Dosage form 13.87 µg AM, 10 µg PE, and 2.5 µg ID	AM			PE			ID		
	Direct method	Found amount^a^	% R^a^	Method	Found amount^a^	% R^a^	Method	Found amount^a^	% R^a^
		14.06 ± 0.05	101.37 ± 0.33	2nd der.	10.06 ± 0.03	100.62 ± 0.29	1st der.	2.52 ± 0.02	100.65 ± 0.66
				RD	10.08 ± 0.02	100.82 ± 0.19	RD	2.51 ± 0.01	100.25 ± 0.02
				DW	10.15 ± 0.06	101.53 ± 0.60	DW	2.54 ± 0.02	101.47 ± 0.78
**Standard addition**	**Direct method**			**DW method**			**DW method**		
	Added amount	Found amount^a^	% R^a^	Added amount	Found amount^a^	% R^a^	Added amount	Found amount^a^	% R^a^
	4	3.96 ± 0.05	98.96 ± 1.13	10	9.97 ± 0.06	99.68 ± 0.60	2	1.99 ± 0.02	99.84 ± 0.98
	10	9.92 ± 0.05	99.22 ± 0.45	40	39.90 ± 0.12	99.75 ± 0.30	5	5.09 ± 0.04	101.86 ± 0.78
	20	20.31 ± 0.05	101.56 ± 0.23	80	78.75 ± 0.12	98.44 ± 0.15	15	14.98 ± 0.05	99.87 ± 0.35

a Average of three determinations

### Dissolution monitoring of the single pill triple therapy

**Fig. 10:**
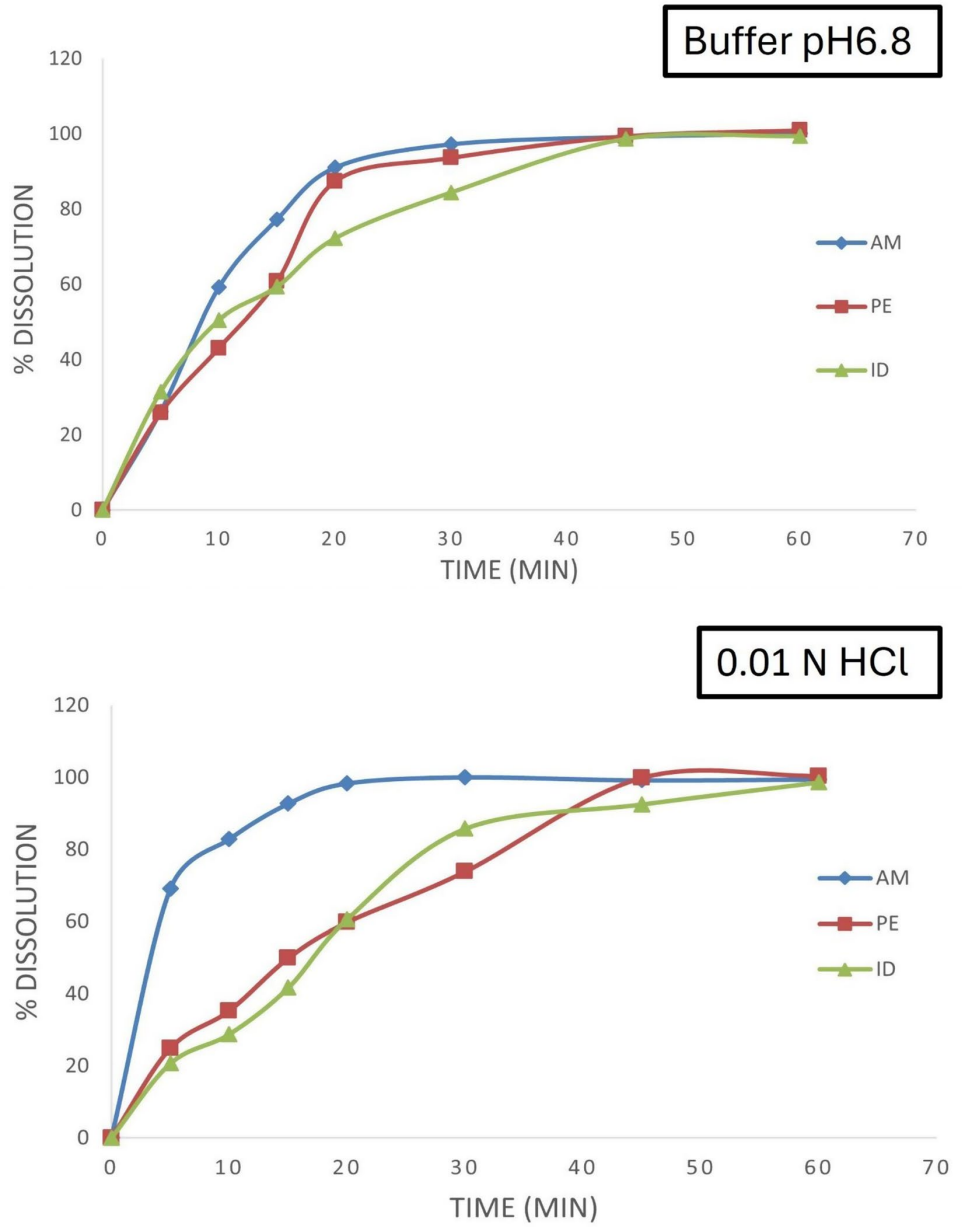
Dissolution profiles for the single pill triple therapy of Amlodipine, Perindopril and Indapamide in phosphate buffer pH 6.8 and in 0.01 M HCl

Dissolution monitoring of AM, PE and ID were performed simultaneously. AM concentrations were determined by direct measurement of the absorbance at 365 nm. Among the three proposed spectrophotometric methods for PE and ID, DW was preferably selected over the other methods for dissolution studies due to its straightforward data processing with acceptable results. The dissolution profiles for the three drugs were assessed in both 0.01 M HCl and phosphate buffer (pH 6.8), resulting in two separate curves being generated for Triplixam^®^(Fig. 10).

### Evaluation of the whiteness of the proposed spectrophotometric method in comparison to various reported methods

**Fig. 11:**
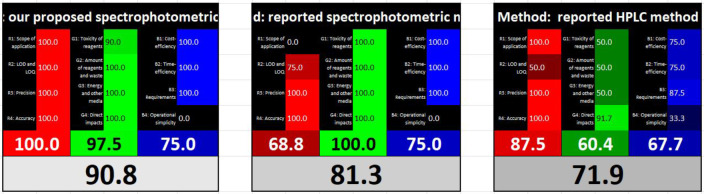
A comparison of three different methods, including our proposed spectrophotometric approach for the determination of AM, PE, and ID, was conducted in accordance with the 12 principles WAC. The analysis was performed using the RGB 12 algorithm

White analytical chemistry (WAC) serves as an extension of the earlier concept of Green Analytical Chemistry (GAC) by incorporating environmental (Green), analytical (Red), and practical (Blue) aspects. This integration replaces the original 12 principles of GAC with the newly formulated 12 principles of WAC. The evaluation of analytical methods is performed using a straightforward RGB 12 algorithm. To conduct the evaluation, users must complete three tables provided in an Excel template, which facilitates the assessment and comparison of different methods. Scores ranging from 0 (least fitting) to 100 (most fitting) should be entered into the designated grey columns. Once the data is entered, the results are automatically computed and displayed in tabular form. Figure 11 presents comparison tables for our proposed method alongside two previously published methods: the sole reported spectrophotometric method [11], one of the reported HPLC method [8]. The results indicate that our proposed method offers an excellent “white” alternative to these previously established methods.

## Conclusion

In this study, we successfully developed and validated simple, diverse univariate spectrophotometric methods to determine AM, PE, and ID in bulk powder, Triplixam^®^ tablets and in dissolution media. These methods offered efficient, eco-friendly, and accurate alternatives for the simultaneous analysis of the ternary mixture. Among the methods, the DW method emerged as the simplest particularly for dissolution studies of the triple pill therapy. The proposed approaches were applied successfully in various dissolution media, proving their practical utility in pharmaceutical analysis. The simplicity, precision, and applicability of the developed spectrophotometric methods together with incorporating WAC principles make them highly suitable for eco-friendly routine quality control of the tablet dosage form. This work also highlights the potential of spectrophotometric techniques as viable tools for dissolution testing, expanding their application beyond drug determination and offering a more accessible approach for in-vitro/in-vivo correlation studies in pharmaceutical research.

## Electronic supplementary material

Below is the link to the electronic supplementary material.

Supplementary Material 1

## Data Availability

The corresponding author can provide the data upon a reasonable request.

## References

[1] Zhang H, Yu LX. Dissolution testing for solid oral drug products: theoretical considerations. Am Pharm Rev. 2004;7(5):26–30.

[2] Dissolution Methods; U.S, Food, Administration D. [https://www.accessdata.fda.gov/scripts/cder/dissolution/dsp_SearchResults.cfm]

[3] British, Pharmacopeia. vol. 1&2. London: Her Majesty’s, stationary office; 2013.

[4] Hoffman BB. Goodman & Gilman’s the pharmacological basis of therapeutics. 11 ed. edn. New York: McGraw-Hill; 2006.

[5] Jackson EK. Goodman & Gilman’s the pharmacological basis of therapeutics. 11 ed. edn. New York: McGraw-Hill; 2006.

[6] Patel K, Chhalotiya U, Kachhiya H, Patel J, Shah D, Nagda D. Simultaneous quantification of Perindopril Erbumine, Indapamide and Amlodipine Besylate in newer combination of anti-hypertensive drugs in Pharmaceutical Dosage Form by Thin Layer Chromatography Method. Sep Sci Plus. 2020;3(5):1–10.

[7] Rezk MR, Badr KA. Determination of amlodipine, indapamide and perindopril in human plasma by a novel LC–MS/MS method: application to a bioequivalence study. Biomed Chromatogr 2021, 35(5). 10.1002/bmc.5048 33314205

[8] Chaudhary BR, Dave JB. Estimation of Perindopril Arginine, Indapamide and Amlodipine in Bulk and fixed dose combination using Stability indicating reverse phase high-pressure liquid chromatography. Int J Pharm Sci Res. 2020;11(12):6267–78.

[9] Metwally MB, Khater DF, Abu-Nameh ESM, AlRashdan Y, Qaisi AM, Salim M. Simultaneous determination of Indapamide, Amlodipine Besylate and Perindopril Arginine Combined in Tablet Dosage Form using high performance liquid chromatography. Jordan J Pharm Sci 2020, 13(4).

[10] Patel KP, Chhalotiya UK, Kachhiya HM, Patel JK. A new RP–HPLC method for simultaneous quantification of perindopril erbumine, indapamide, and amlodipine besylate in bulk and pharmaceutical dosage form. Future J Pharm Sci 2020, 6(80).

[11] Alves E, Nazareth C, Pereira S. Development and validation of a Novel UV Spectrophotometric Method for Simultaneous Analysis of Amlodipine, Indapamide and Perindopril. Indian J Pharm Sci. 2020;82(5):843–50.

[12] Horváth IT, Anastas PT. Innovations and Green Chemistry. Chem Rev. 2007;107(6):2169–73. 17564478 10.1021/cr078380v

[13] Kirchhoff MM. Promoting Green Engineering through Green Chemistry. Environ Sci Technol. 2003;37(23):5349–53. 14700319 10.1021/es0346072

[14] Nowak PM, Wietecha-Posłuszny R, Pawliszyn J. White Analytical Chemistry: an approach to reconcile the principles of Green Analytical Chemistry and functionality. TrAC Trends Anal Chem. 2021;138:116223.

[15] Zaazaa HE, Elzanfaly ES, Soudi AT, Salem MY. Application of the ratio difference spectrophotometry to the determination of Ibuprofen and Famotidine in their combined dosage form; comparison with previously published spectrophotometric methods. Spectrochim Acta Part A Mol Biomol Spectrosc. 2015;143:251–5. 10.1016/j.saa.2015.02.050 25733252

[16] Zaazaa HE, Elzanfaly ES, Soudi AT, Salem MY. Spectrophotometric Method for the determination of Two Coformulated Drugs with highly different concentrations. Application on Vildagliptin and Metformin Hydrochloride. J Appl Spectrosc. 2016;83(1):137–40.

[17] Boltia SA, Soudi AT, Elzanfaly ES, Zaazaa HE. Simultaneous Determination of Paracetamol, Orphenadrine Citrate, and Caffeine Ternary mixture by different spectrophotometric methods. J Appl Spectrosc. 2019;86(4):668–661.

[18] Soudi AT, Hussein OG, Elzanfaly ES, Zaazaa HE, Abdelkawy M. Simultaneous determination of Phenazopyridine HCl and Trimethoprim in Presence of Phenazopyridine HCl impurity by Univariate and Multivariate Spectrophotometric methods - quantification of phenazopyridine HCl impurity by Univariate methods. Spectrochim Acta Part A Mol Biomol Spectrosc. 2020;239:118516118511–118510. 10.1016/j.saa.2020.118516 32492634

[19] Merey HA, Ramadan NK, Diab SS, Moustafa AA. Spectrophotometric methods for simultaneous determination of Ternary mixture of Amlodipine besylate, Olmesartan medoxomil and hydrochlorothiazide. Spectrochim Acta Part A Mol Biomol Spectrosc. 2014;125:138–46. 10.1016/j.saa.2014.01.095 24534425

[20] Al–Alamein AMA, El–Rahman MKA, Fawaz EM, Abdel–Moety EM. Uv–Spectrophotometry Versus HPLC-PDA for dual–drug dissolution profiling: which technique provides a closer step towards Green Biowaiver Concept? Novel application on the recent FDA–Approved mixture Aleve Pm. Chem Pap. 2018;73:309–19.

[21] United States Pharmacopeia and National Formulary (USP 37-NF 32.). Rockville: United States Pharmacopeial Convention; 2014.

[22] Anand O, Yu LX, Conner DP, Davit BM. Dissolution testing for generic drugs: an FDA Perspective. AAPS J. 2011;13(3):328–35. 21479700 10.1208/s12248-011-9272-y PMC3160163

[23] Borman P, Elder D. Q2(R1) Validation of Analytical Procedures. In: *ICH Quality Guidelines.* Edited by Teasdale A, Elder D, Nims RW; 2017: 127–166.

