# Supplementary figures and images for: Application of spectrophotometry in novel simultaneous dissolution profiling of a single pill triple therapy of amlodipine, perindopril and indapamide; whiteness evaluation
Source: BMC Chem. 2025 Feb 7;19(1):33. doi: 10.1186/s13065-025-01396-3 (PMC11806599; doi:10.1186/s13065-025-01396-3)

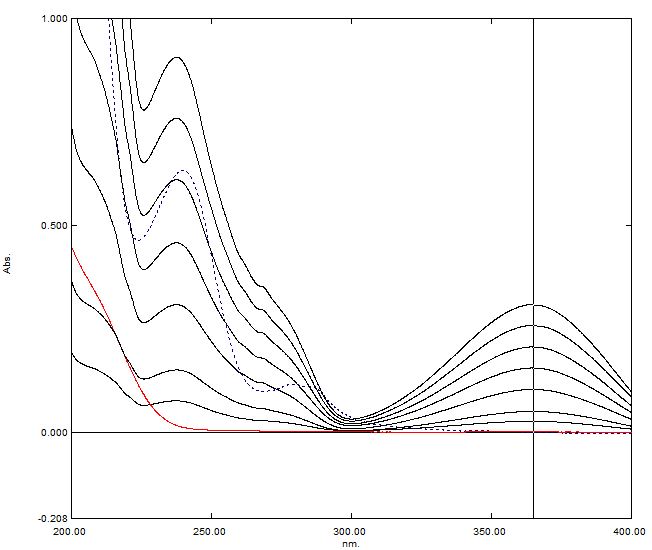


Figure S1: calibration of AM at 365 nm with zero PE and ID.

Supplement: Supplementary file 1 — Supplementary Material 1 [file 13065_2025_1396_MOESM1_ESM.docx]
